# Complete genome sequence of *Bifidobacterium longum* subsp. *longum* strain iVE-15

**DOI:** 10.1128/mra.00202-26

**Published:** 2026-07-10

**Authors:** Mallory J. Van Haute, Katherine Chacón-Vargas, Shara R. P. Yumul, Chloe M. Christensen, Robert Hutkins, Thomas A. Auchtung

**Affiliations:** 1 Synbiotic Health, Inc., Lincoln, Nebraska, USA; 2 Department of Food Science and Technology, University of Nebraska-Lincoln 14719, Lincoln, Nebraska, USA; 3 Nebraska Food for Health Center, University of Nebraska-Lincoln 822544, Lincoln, Nebraska, USA; Wellesley College, Wellesley, Massachusetts, USA

**Keywords:** *Bifidobacterium longum*, iVE-15, carbohydrates, B vitamins, cardiovascular health, immune health

## Abstract

*Bifidobacterium longum* subsp. *longum* is a gastrointestinal commensal associated with positive effects on host health. Here, we announce the complete genome sequence of human isolate *Bifidobacterium longum* subsp. *longum* iVE-15 that is predicted to encode for multiple beneficial traits, including carbohydrate utilization, vitamin biosynthesis, cardiovascular support, and immunomodulatory activity.

## ANNOUNCEMENT

*Bifidobacterium longum* subsp. *longum* is a common (1) member of the human gut microbiota for which some strains have been demonstrated to improve host metabolic (2), gastrointestinal (3), respiratory (4), immune (5), and/or mental (6) health. *Bifidobacterium longum* subsp. *longum* iVE-15 (formerly CR15) was previously isolated following *in vitro* enrichment (IVE), a strategy to select for potentially synergistic probiotic strains (7). Briefly, the donated fecal sample of a healthy adult in Nebraska was inoculated into 37°C anoxic fermentation medium (8) containing xylooligosaccharides (XOS) as the only carbohydrate. The culture was successively transferred every 24 h into fresh medium, then after 96 h, was plated on *Bifidobacterium*-selective agar. One isolate, iVE-15, was shown to establish in 18/20 fecal communities when re-introduced in the presence of XOS (7). A draft genome of iVE-15 was previously sequenced (NCBI Assembly ASM643897v1).

iVE-15 from an original freezer stock was cultivated overnight in anoxic MRS broth (BD Difco) at 37°C and harvested in late exponential phase by centrifugation. CD Genomics (Shirley, NY, USA) performed DNA extraction using the DNeasy UltraClean Microbial kit (Qiagen) according to the manufacturer’s instructions. DNA was sheared to 10 kb using a g-TUBE, purified, end-repaired, and ligated to form a SMRTbell (v3.0) library. The qualified library was sequenced on a PacBio Revio (v2 chemistry), and data was processed with SMRT Link v8.0.

The 298,568 HiFi reads (mean length 7,022 bp; *N*_50_ = 7,580 bp; median Q41) were corrected and trimmed using Canu (v2.1.1), assembled, and overlap trimmed with Flye (v2.9.1). Rotation was not performed. The complete genome of iVE-15 is composed of a 2,441,751 bp circular chromosome (60.39% GC) and one 6,985 bp plasmid (62.81% GC). Annotation by Prokka (9) identified 2,005 protein-coding sequences, 57 tRNA, 8 rRNA, and 1 tmRNA. Additional annotation was performed using eggNOG-mapper v2 (10, 11), CRISPRCasFinder (v1.1.0) (12), and BAGEL4 (13). The taxonomic classification of iVE-15 was confirmed by using Snippy (v4.6) (14) and FastTree (v2.1) (15) with publicly available genomes of *B. longum* to generate a SNP-based phylogeny. In addition, pairwise ANI was calculated between iVE-15 and other genomes using FastANI (v1.32) (16). Values were ≥98.2% to *B. longum* subsp. *longum* but ≤97.0% to other subspecies. Default parameters were used for all software.

**Fig 1:**
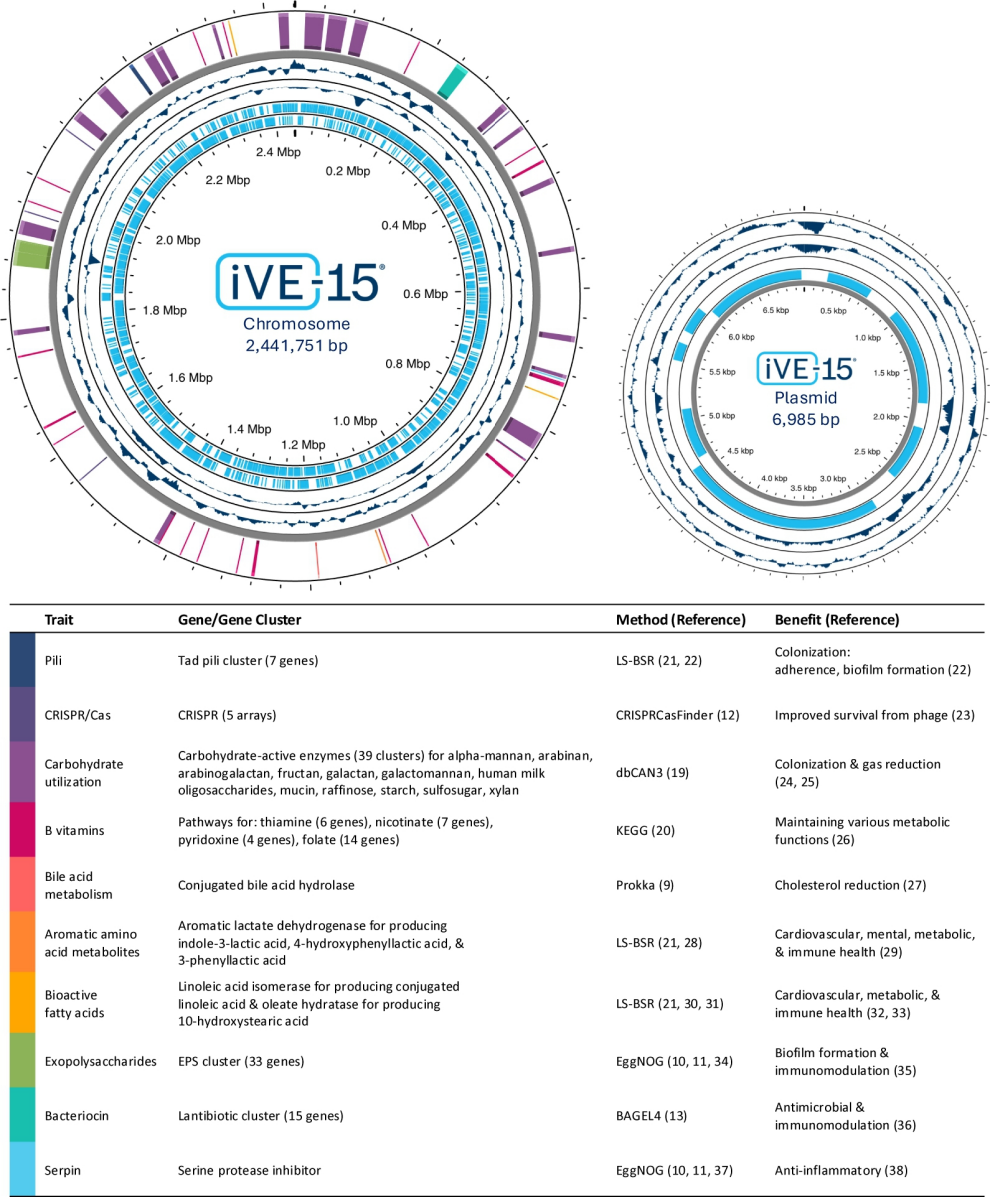
Map of *Bifidobacterium longum* subsp. *longum* iVE-15 chromosome and plasmid. From the inner to outer circle are the following: tick marks (Mbp/kbp), CDS reverse strand (light blue), CDS forward strand (light blue), GC content (dark blue), GC skew (dark blue), and genes associated with potential host health benefits (pili, dark blue; CRISPR/Cas, violet; carbohydrate utilization, purple; B vitamins, magenta; bile acid metabolism, peach; aromatic amino acid metabolites, orange; bioactive fatty acids, yellow; exopolysaccharides, green; bacteriocin, turquoise; serpin, light blue). The figure was generated with Proksee (9–13, 19–39).

Genes and pathways relevant to potential human health benefits were examined (Fig. 1). Analysis using CARD RGI (17) and VFDB (18) detected no antibiotic resistance or virulence genes. The iVE-15 genome encodes numerous genes implicated in stress resistance and colonization mechanisms. Analysis by dbCAN3 (19) identified 39 putative carbohydrate-active gene clusters for 12 CAZyme substrates. KEGG (20) annotation indicated that iVE-15 harbors pathways for B vitamin metabolism, including thiamine (B1), nicotinate/nicotinamide (B3), pyridoxine (B6), and folate (B9). Genes relevant to cardiovascular health were present, including those for bile metabolism and production of aromatic amino acid metabolites and bioactive fatty acids. Additionally, multiple genes relating to host immunomodulation were identified, including the production of exopolysaccharides, a lantibiotic, and a serpin. In summary, the iVE-15 genome encodes numerous traits with potential to benefit human health.

## Data Availability

The iVE-15 BioProject, BioSample, SRA, and GenBank complete assembled genome accession numbers are PRJNA1348473, SAMN54979378, SRR35887913, and JBUCOC000000000.
